# Are Most Women Crazy?

**Published:** 1880-12

**Authors:** 


### ARE MOST WOMEN CRAZY?

When the Board of Managers of the Buffalo State Asylum for the Insane, was discussing the question as to whether that institution should be opened for men or women, the President stated, in effect, "that he could fill the wards with female patients in three months." It is with a feeling of sadness that we hear this announcement from one whose experience with the gentle sex has been varied and extensive. There are so many women who seem to take solid comfort in the nursing of some pet ailment that it would not be surprising if the average physician looked upon a large part of them as mentally "a little off." But he is an unfortunate one indeed who can, at short notice offer, to stock a whole asylum, and we venture to hope that such an estimate of the degeneracy of the feminine mind has been intentionally (or otherwise) exaggerated.

